# Coronavirus versus the textile industry: cluster lessons for future challenges

**DOI:** 10.1186/s40691-021-00284-3

**Published:** 2022-03-25

**Authors:** Francisco Puig, Santiago Cantarero, Francesco Verdone

**Affiliations:** Department of Management, Faculty of Economics, University of Valencia, Valencia, Spain

**Keywords:** Covid-19, Clustering, Crisis, Strategic response, Valencian Textile Cluster, Spain, F23, L67, R11, R12

## Abstract

Since the pandemic situation was officially declared, part of society was immersed in searching desperately for solutions to combat it. Textile firms addressed with uneven effectiveness the challenge of creating products that helped medical and civil professionals (e.g., personal protection equipment, masks, sanitary material, etc.). To do this, they had to face supply problems, lockdown, and make a significant innovative effort. This work aims to analyze the strategic response of the companies belonging to the Valencian Textile Cluster (VTC) (Spain) to the coronavirus crisis and the territorial factors that influenced it. We carried out a content analysis of the news in the main newspapers during February–July (2020). Our analysis revealed that, on average, VTC firms responded to the challenge more quickly and effectively than other Spanish textile firms. The most influential location-specific factors were the clustering developed, institutional support, and a deep-rooted tradition in producing technical-home textiles, although we also detected that social media collaborated in the process of transferring value information. The influence of all these factors was more intense in the epicenter of the cluster (Ontinyent). Consequently, our results highlight the cluster effect and offer lessons that can help manage unexpected future events more effectively.

## Introduction

*"During the pandemic, hospitals in the area ran out of supplies, and textile companies provided blankets and pajamas for patients. These companies also reinvented themselves by launching sanitary products that they donated to us (masks, gowns, non-woven fabric, waterproof fabrics, and materials of all kinds). For us (the health workers), that quick, determined, and effective action in those moments of stress, insecurity, and fear of those first days will never be forgotten".*

Margarita Llaudes. Directora del área de Salud de Xàtiva-Ontinyent

When the pandemic coronavirus was announced in early 2020 and, among other things, production at Chinese textile companies was halted, global supply chains were starved for textile materials and essential products (yarns, fabrics, and other goods) (Gereffi, 2020). In a few weeks, the risks of globalization and external dependence for manufacturing textile products became evident. For example, the fashion giant Inditex was almost paralyzed due to its suppliers' shortage of raw materials and supply problems (Dowsett, 2020). The supply chain disruption also affected other textile firms that had to buy the materials from different suppliers located more closely (McMaster et al., 2020). The alarm was raised regarding the limited capacity to respond and produce products that would help to combat the pandemic (e.g., personal protection equipment, masks, sanitary material, etc.). The testimony of Margarita Llaunes (Llaunes, 2020) (written just below the title of this section), who is responsible for health services in a significant geographical area occupied by some 200,000 inhabitants, serves to illustrate this statement.

Expressly, when on March 11, 2020, the World Health Organization (WHO) officially declared the spread of the COVID-19 pandemic (WHO, 2020), society immediately found itself in a race against time to fight the virus more effectively. On the one hand, scientists studied the virus and searched for a vaccine that would halt it. On the other hand, we witnessed homemade inventions, social initiatives, and an exemplary rapid and innovative response from the textile industry. It strived to meet this challenge of protecting the population. Moreover, when the lockdown was declared, some textile areas, far from closing the factories and ceasing production, began to carry out intense and innovative activities, working together in solidarity to respond to the needs of doctors and nurses who needed essential supplies (Belso-Martinez et al., 2020). The challenge was enormous: Global supply chains were failing; there were no materials, and there was no machinery for production (Gereffi, 2020).

Authors such as Chen (1996) suggest that the firm's strategic response depends on a set of critical organizational determinants that would cause companies immersed in a similar situation to differ in identifying the problem and the strategies to implement through their AMC's model (Awareness-Motivation-Capability). Wenzel et al. (2020) have analyzed the types of responses/strategies companies adopt in times of crisis along the same lines. In both studies, it is assumed that the firm-specific factors (size, ownership, activity, among others) explain the different implemented decisions. However, they do not consider other location-specific factors that would also influence these decisions, such as being located in an industrial cluster (Fromhold-Eisebith et al., 2021). Regional clusters, localized production systems, or industrial clusters can be understood as a territorial organizational model characterized by a geographic agglomeration of interconnected organizations (firms and institutions) in related industries (Porter, 1998).

Today, we can see that the response against the coronavirus was not the same in all territories. For example, while the Valencian textile cluster (VTC) effectively innovated and continuously produced masks, personal protection equipment (PPE), and other sanitary textile products (El Periodic, 2020), other important textile Spanish regions (as Catalonia or Galicia) did not register this positive evolution (Gutierrez, 2021; Sío, 2020). For all this, important questions arise, such as what has been the cluster's role on the strategic response of these firms.

Therefore, the objective of this work is to analyze the response of the VTC's firms to the coronavirus crisis and the territorial factors that influenced it. For this, we carried out a content analysis of the news that appeared in the leading newspapers during February–July (2020). According to Wenzel et al. (2020), our work concludes that in the VTC, the strategic response can be labeled as mainly innovative. The clustering and a deep-rooted tradition in producing home-technical textiles influenced it, being more intense at the cluster's epicenter (Ontinyent). The results of this study have important strategic, policy, and socio-economic implications. Strategic because assessed the spatial relationship between crisis and firm's response. For policymakers can serve as a guide to be more effective when facing a crisis. For people interested in the textile industry, we present valuable lessons to evaluate the socio-economic importance of that sector.

This paper is organized as follows: Sect. 2 describes the Spanish textile-clothing industry and summarizes the study's theoretical background. Section 3 describes the data and methodology. Section 4 presents and analyzes the results. The last section summarizes the main conclusions of the study.

## Literature review

### The textile industry in Spain: relevance and location.

The textile-clothing industry is one of the main economic sectors, and it is regarded as one of the traditional manufacturing industries. The European Commission (2020) defined the textile industry as belonging to a set of diverse and heterogenic industries that produce a wide variety of products, from synthetic yarn derived from high-technology to woolen fabrics, cotton bed linen, industrial filters, nappies, and haute couture. This final product diversification is correlated with a copious amount of industrial processes, companies, and market structures.

Besides the important economic effect of this industry in Europe (more than 160,000 companies), it is impossible to deny the social and strategic value. The 2019 novel coronavirus pandemic (COVID-19) has determined the value of products and their functions; the security of employees in the healthcare system, people in general, and those who depend on the industry. Without any doubt, the importance of it and its influence on the economic sphere has increased significantly. According to the Eurostat Report (2020), it is impossible to construct cars, planes, buildings, agricultural equipment, and military machines for defense and security without textile materials. Moreover, artisans cannot undertake their work.

The textile-clothing sector is heterogeneously dispersed geographically, diverse and different activities and industries form it. In this paper, we used the classification proposed by Puig et al. (2009), which identified the following five basic subsectors: Yarn; Finished products; Home-technical; Knitted textiles, and Clothing. This classification has significant advantages. The first is that it allows us to separate production from commerce. The second advantage is that it is more operational when compared with other commonly used classifications (e.g., the classification based on yarns, fabrics, made-up textile products, and Clothing). The third advantage concerns the isolation of other related sectors supporting textile machinery or textile chemistry. According to this classification, most sanitary textile articles (e.g., bed linen, bath linen, dining room linen, and uniforms) and other sanitary products (e.g., gowns, gloves, masks, surgical drapes, and garments for wound care, incontinence management, and hygiene) are included in the Home-technical subsector.

According to Eurostat (2020), the European textile-clothing industry accounts for around 9% of manufacturing employment (1,664,000 employees), of which 33% is attributed to companies dedicated to textiles and 67% to clothing articles. The main countries involved in the production include Italy, Germany, Spain, France, and Poland, which account for approximately 63%. On average, each firm employs around 20 employees.

An essential characteristic of this industry is that it tends to be geographically concentrated in a non-random manner, showing specific productive specialization patterns in certain regions (Stengg, 2001). Thus, while Germany and France are more focused on the Textile industry and have more capital-intensive companies, Spain, Italy, and Poland are more heavily focused on Clothing and labor-intensive activities. Another key characteristic is the evolution that, on average, this industry has followed: It has registered a net decline of 15%, being countries such as Poland, Romania, and the Czech Republic among the few countries that have recorded significant growth.

The textile sector in Spain shows similar structural characteristics to the European industry, i.e., productive specialization, geographic agglomeration, small-sized companies, and uneven development. For example, while there are around 55,800 Spanish textile companies (SABI, 2020), 42% are dedicated to textiles, and 58% are associated with Clothing and fashion. The following brands stand out: Zara, Massimo Dutti, Bershka, Pull&Bear, Stradivarius, and Mango y Desigual (Miranda, 2020).

**Fig. 1:**
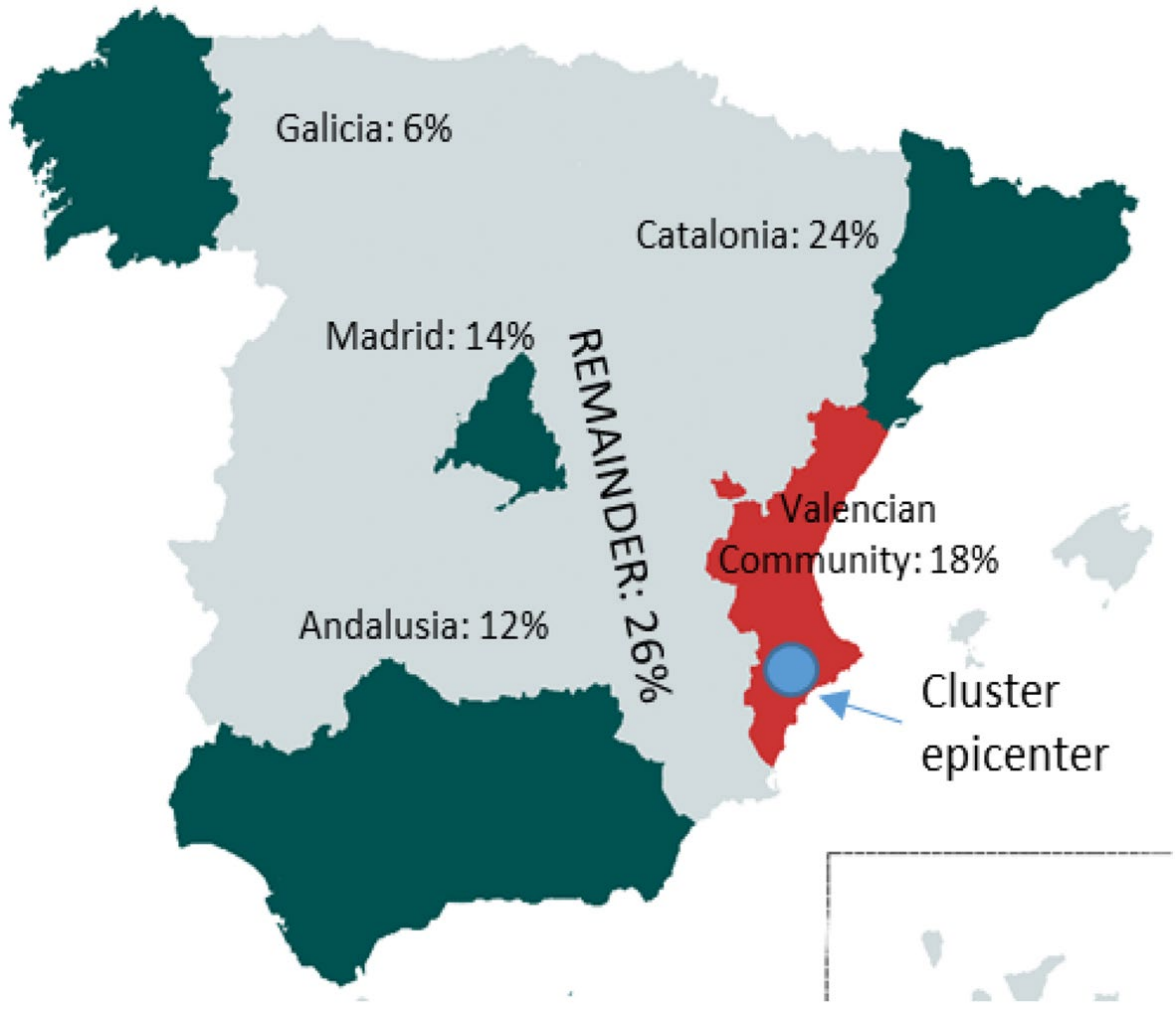
Geographical concentration of the textile-clothing industry in Spain in 2020. The percentages indicate how many firms are located in each area. Valencian Community agglomerated 18% of the total. The data were obtained from SABI; the map and representation are self-made

Moreover, remarkably, this industry is located mainly in Catalonia (24%) and the Valencian Community (18.5%), followed by Andalusia (12%), Madrid (14%), and Galicia (6%), being shared the remainder 26% on rest of the Autonomous Communities of Spain. (Fig. 1). The Valencian textile industry differs from other regions in Spain because most of the industry is concentrated in a relatively specific geographical area known as the Valencian Textile Cluster (VTC). The epicenter of which is Ontinyent (Puig y Marques, 2010), and it is focused on the Home-technical textile subsector (33% concentration of Spanish production). The VTC is home to around 880 companies, and it directly employs 13,200 people (SABI, 2020). It contains two universities, a textile training center, one textile business association (ATEVAL), and one research institute (AITEX), among other institutions.

Different academic papers have analyzed the VTC as an example of resilience, competitiveness, and model of territorial organization (Puig et al., 2013). For example, the research studies carried out by Capó-Vicedo et al. (2007), Puig et al. (2009), and Belso-Martínez et al. (2019) highlighted the international competitiveness of the model of industrial organization, outlining its efficiency for creating the front for globalization. Other authors, such as Belso et al. (2011), Molina-Morales et al. (2012), or Camarena et al. (2020), have studied how the physical and social proximity between their participants facilitated clustering, which strengthened the sense of belonging, encouraged the exchange of information and knowledge, and influenced the innovation activity in the textiles firms located in the VTC. Similarly, Expósito-Langa et al., (2015a, b) have analyzed how the shared vision and networking intensity affect the innovation performance of these firms.

The VTC has recently become the subject of numerous debates because from March to July 2020, it fought against the pandemic and Covid-19 (Belso et al., 2020). As the president of the principal association of the sector (ATEVAL), Pepe Serna declared, "the satisfaction is that the textile sector (Valencian) has known how to react to the challenge of the coronavirus." (El Nostre Ciutat, 2020) In the same vein, other comments were published in different media outlets by the president of the Generalitat Valenciana and the Conseller of Economy.

As evidenced by the various forms of news media (TV, radio, newspapers), the VTC readapted and reconfigured its production processes to manufacture masks, gowns, and gloves during a brief period, which helped citizens protect themselves from the virus. This capacity for change caused the region to transform towards sanitary and other textile products and survive the economic crisis arising from the pandemic. It manufactured from zero to 55 million masks per month in just three months, and it currently exports a proportion of its PPE to countries including India, Colombia, and Mexico (El Nostre Ciutat, 2020). Valencian firms like Textils Mora, Virus Protec, and Airnatech have been noticed because they innovated and registered new products to combat the pandemic (mantaescola, and biocide, and antiviral plus masks, respectively). Recently, some cluster firms began to collaborate on critical public tenders (at a regional and national level and with the United Nations).

What was the response of the Valencian textile companies to combat the pandemic, and why was it different from that of other Spanish textile companies? In line with the main body of literature publications concerning firm strategy, competitiveness, and crisis, we believe that the answer to these questions lies in the clustering developed to turnaround the situation and territory conditions.

### The competitiveness and Spanish textile industry's location-specific factors

Firm competitiveness describes the capability of a firm to sustainably fulfill its dual purpose, i.e., meeting customer requirements at a profit (Chikán, 2008). Competitiveness is also the ability to compete. This capacity or ability depends on a set of determinant factors that are, in a geographic context, situated closer to the activity. Michael Porter (Porter, 1990) identified the following relevant determinants: Factor conditions, demand conditions, home market rivalry, related and supporting industries. These determinants are mutually influential, wherein each influence and are influenced by other determinants. The mechanisms through which this mutual interrelationship is exercised are termed clustering.

In works as Cluster Competitividad (1999) or Payeras et al. (2001), the determinant factors of the VTC have been analyzed. In these papers, the "conditions of the factors" refer to human resources, physical resources, knowledge resources, capital resources, and infrastructures available in the territory, mainly in the cities of Valencia, Alicante, Alcoi, and Ontinyent (for example, wastewater treatment plants, broad and effective training facilities, research centers, and business associations). The "conditions of the demand" refer to the nature of domestic demand for products or services in the sector. In this regard, one of the main elements of this factor includes the dynamism and impulse associated with companies' innovations. "Related and support sectors" refer to the presence of supplier sectors and related sectors in the territory that are effective and efficient, provide the necessary inputs, and produce in the best possible way (e.g., textile machinery repair shops, chemical industry, or import–export service companies). Finally, "the company's strategy, structure, and rivalry" refer to how companies and domestic rivalry are created, organized, and managed in each territory. The advantages are garnered from a good fit between the modes of organizing companies and the goals and sources of the competitive advantages of a sector. Some examples of this determinant's influence include a strategy that aims to develop distribution channels or cooperation channels, that allows textile companies to enjoy greater powers of negotiation in the territory, or that facilitates the undertaking of R&D projects. Finally, the role of chance or luck, and the role of governments, should be added to these four determinants to explain how the model works. Porter's model has been widely employed by different types of research that examined the territory and the competitiveness of textile companies: Giuli (1997), at the level of the EU; Jin and Moon (2006), for Korea; Shafaei (2009), in Iran; or Cluster Competitiveness (1999), for the Valencian Textile Cluster.

The cluster's existence is a necessary condition, albeit insufficient, for this extra competitiveness to be significant (Camarena et al., 2020). It also requires effective clustering, understood as the mechanism by which the actors, companies, people, and institutions interact. When all of this occurs, it is possible to identify three effects of the cluster: It increases entrepreneurial activity, improves efficiency rates, and facilitates innovation processes (Delgado et al., 2010).

For the case of the VTC, this clustering generates a business and knowledge dynamic that facilitates the growth and development of these participating companies, acting as an attraction element for other companies and actors (Belso-Martínez et al., 2006; Molina-Morales et al., 2012). Also, the clustering has facilitated trust, information diffusion, and saving in costs (b; Expósito-Langa et al., 2015a). And from a strategic perspective, it influenced the response to face the crisis that in 2005 these firms were immersed in the interpretation of the situation, the motivation to act, and the ability to do it (Pla-Barber et al., 2007).

### Textile cluster against coronavirus crisis

According to Wenzel et al. (2020), in times of crisis (such as the COVID-19 pandemic) and when survival is threatened, firms can implement four generic types of different responses: Retrenchment, Persevering, Innovating, and Exit. The decision for one or another depends on multiple internal and external factors (Kraus et al., 2020). For example, retrenchment involves the sale of some business units, the partial closure of activities, and workforce reductions for a textile firm. The ultimate objective of these measures is to reduce costs and obtain liquidity to tackle the situation (Robbins & Pearce, 1992). Persevering means that a company chooses to persevere in its activity without making notable changes to confront the problem in the best way possible, hoping that things will change one day. When a firm opts for the Exit strategy, it has decided to implement the toughest of all decisions; closure. When firms deployed this decision is because they believe that they cannot recover the situation. It is important to note that some threatened companies that were interconnected with the textile industry chose to adopt one of the first three types of measures aforementioned, so it can be said none of which proved successful in the fight against the pandemic.

However, companies that opted for an Innovating response succeeded, and they were able to take advantage of the opportunities that emerged from the crisis. Therefore, Valencian companies such as Textils Mora, Virus Protec, and Airnatech or others like Textiles El Delfín, Rapife, Texting Cleaning Solutions, Euromoda, Textils Mora, Marie Claire, or Cotoblau responded quickly and effectively to the demand for masks, gloves, gowns, and PPE equipment, among other products.

The strategic response of these textile companies can be analyzed by referring to the literature on business turnarounds (Robbins & Pearce, 1992; Schmitt & Raisch, 2013; Rico et al., 2021a): speed and type of strategy, and the effectiveness of the responses implemented. In times of crisis, the rapid identification of causes and implementing responses are crucial to survival (e.g., supply problems and the redirection of production towards sanitary textiles). In line with Chen (1996) and Barker and Duhaime (1997), different factors influence the response to a crisis that can be grouped according to perception from managers about the causes of decline (industry-wide or firm-specific factors) and several organizational attributes (resources and capabilities). For example, Pla-Barber et al. (2007) analyzed the managerial response that Valencia textile firm faced with the crisis provoked by the liberation of market textile in 2005. They evidenced three main postures, one more passive that can be associated with persevering. Another profile linked to the Retrenchment and Exit, and a third characterized for a proactive and combative attitude similar to the described by Wenzel et al. (2020) as Innovative. In this research, firm-specific factors as the education and experience of managers and subsector of activity influenced the decision.

The literature has studied the factors associated with the effectiveness of the responses implemented. Joint with speed in the response and the risks assumed (e.g., lack of liquidity, dismissal of critical employees, or neglect of customers) are location-specific factors as the support and collaboration of the territory's stakeholders (i.e., shareholders, workers, clients, suppliers, banks, unions, and governments) (Rico et al., 2021a, 2021b). According to Porter's model (Porter, 1990), these stakeholders include the local and regional government, research institutes, and other essential institutions (e.g., ATEVAL and AITEX).

In short, to understand at a territorial level the unequal response of similar companies (textile firms) to the coronavirus crisis, it is necessary to note that this industry is heterogeneous (from subsectors labor-intensive to knowledge-intensive) and distinguish the cluster from the clustering. The cluster can be understood as a photograph of the geographic location of the companies within a given territory (necessary condition). At the same time, clustering can be regarded as the mechanism by which actors, who form part of the cluster, interact with each other (i.e., firms, people, institutions). From this perspective, it can be argued two fundamental aspects: a) there are not two identical clusters (Porter, 1998), and b) the cluster does not have a similar effect on all of the companies within it due to their organizational diversity regarding their activities, managers, and firm size (Claver-Cortés et al., 2019). For all this, the cluster answers to the environmental challenges can be different because within the same industry differs the interaction and content of the relationships among the interconnected actors that conform it. It seems to be the case for the Catalonian (Gutierrez, 2021) and the Galician textile (Sío, 2020).

## Methods

This research aims to analyze the response of the VTC's firms to the coronavirus crisis and the territorial factors that influenced it. In line with Belso-Martínez et al. (2020), given the research questions, objectives, and the studied context, we carried out a content analysis of the news that appeared in the leading newspapers during February–July (2020). This methodology has been used by Joshi and Swarnakar (2021) and Shin et al. (2021) to study the social reaction of firms to the COVID-19 crisis. Authors like Islam et al. (2021) have also undertaken a systematic content analysis of articles published in newspapers in the textile industry. In our research, the content analysis has been carried out in Spanish magazines and newspapers (local and national) with news publications related to the textile industry, including in the FACTIVA database, during the period from March to June 2020, coinciding with the period of total confinement of Spain due to COVID 19. For our purposes, the content analysis has the advantage of allowing the identification and classification of the news that appeared in the written press during the lockdown as a proxy of strategic responses.

FACTIVA is an international database with press, financial and corporate information (with more than 35,000 sources) produced by Dow Jones, which provides information for making decisions in companies, investments, and academic analysis (www.dowjones.com). This database has been used in a large number of academic publications related to economics (Monfort et al., 2021), sociology (Kiousis et al., 2007), marketing (Golfetto & Rinallo, 2008), and management (Brammer & Millington, 2006). It has also been used in academic articles related to COVID-19 (Joshi & Swarnakarn, 2021), the textile industry (Islam et al., 2021), and the Spanish textile industry (Belso-Martínez et al., 2020), among others.

Like Joshi and Swarnakar (2021) or Shin et al. (2021), we defined the terms for the search in the FACTIVA database according to the research object of this work. First, "Textile Industry" (and similar terms and synonyms: textile sector, textile cluster, textile companies) is considered an essential item since it is intended to investigate this specific sector. Second, the other basic term is considered: "COVID" (and similar and/or related terms: coronavirus, pandemic, virus). The simultaneous search for the previous basic terms in the same news item or mention published in Spain and collected in the FACTIVA database performance an initial result of 301 articles. After eliminating duplications due to being agency news published in more than one medium or due to repetition between the same business group publications, it gives rise to 51 published news from February to July.

**Fig. 2:**
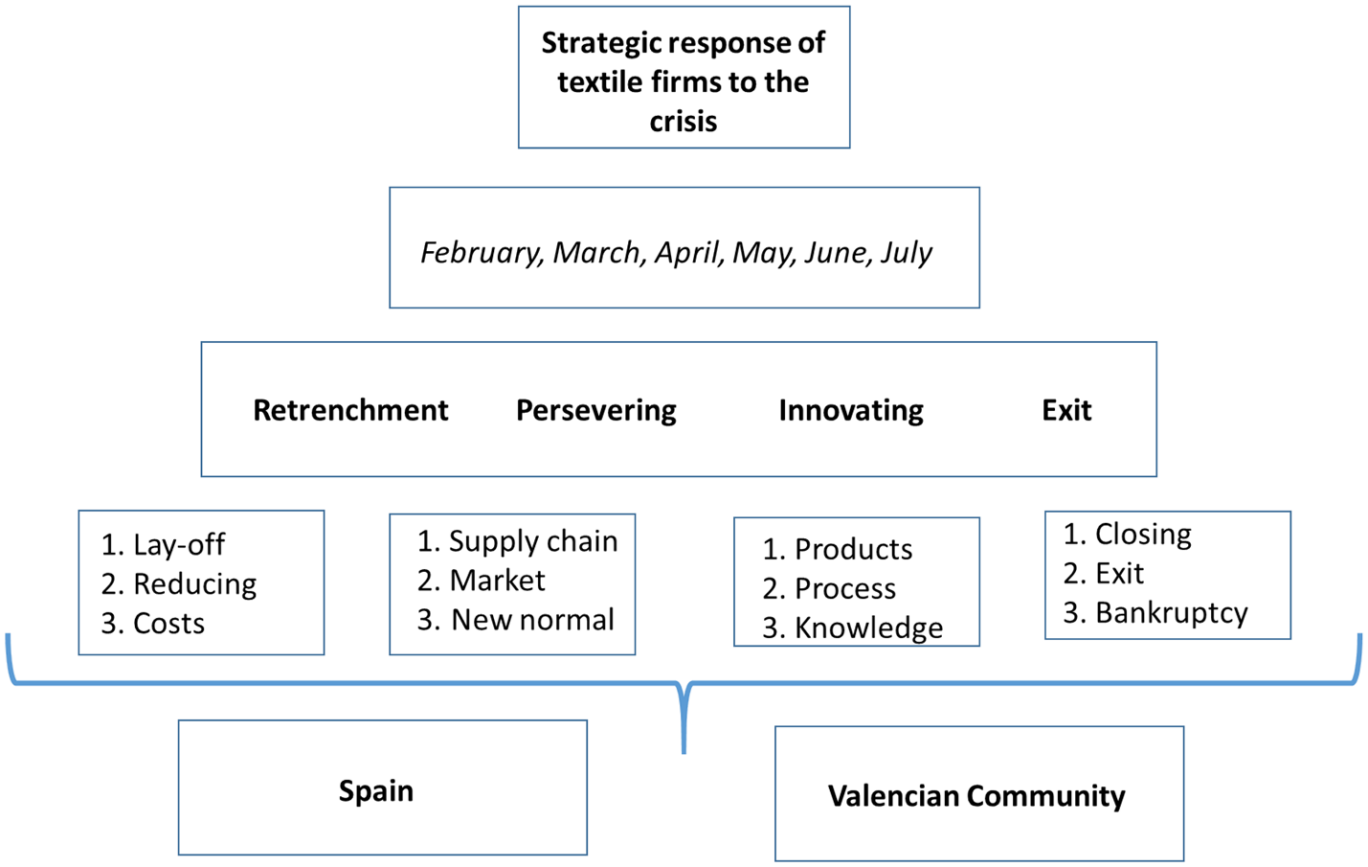
Umbrella research design. The cascade structure goes from more general (strategic response) to more specific (subcodes). The latest level indicates that the codes differentiated the spatial origin of the news

The content of each of the 51 news items was analyzed using the ATLAS.ti tool, a professional software program for qualitative data analysis used worldwide by leading institutions and researchers to professionally analyze texts and multimedia data (Legewie, 2014). The research carried out with ATLAS.ti follows a cascade structure or umbrella design (from more general to more specific) (see Fig. 2) (Strauss & Corbin, 1998) as follows: First, marking the broader concept, which in our case is the "response of textile companies to the crisis." Second, differentiating this behavior in periods, which are the months of publications under study. Below this level are the four situations in which a company can be immersed following the approach already described by Wenzel et al. (2020) (Retrenchment, Persevering, Innovating, and Exit). Finally, in the later levels of the umbrella, distinguishing the subcodes that are part of each code (nine in total) and the spatial code of each news (Spain and Valencian Community). In line with Krippendorff (1990) and Varguillas (2006), the definition of the subcodes that allowed identifying the news that belongs to each main code was developed by two researchers responsible for the study, who obtained the same result. The temporal and geographic coding does not admit doubt since it is expressed objectively in each article reviewed. In the Annex 1: Table 2 shows the relationship between codes and subcodes applied in ATLAS.Ti.

## Results and Discussion

### Analysis

**Fig. 3:**
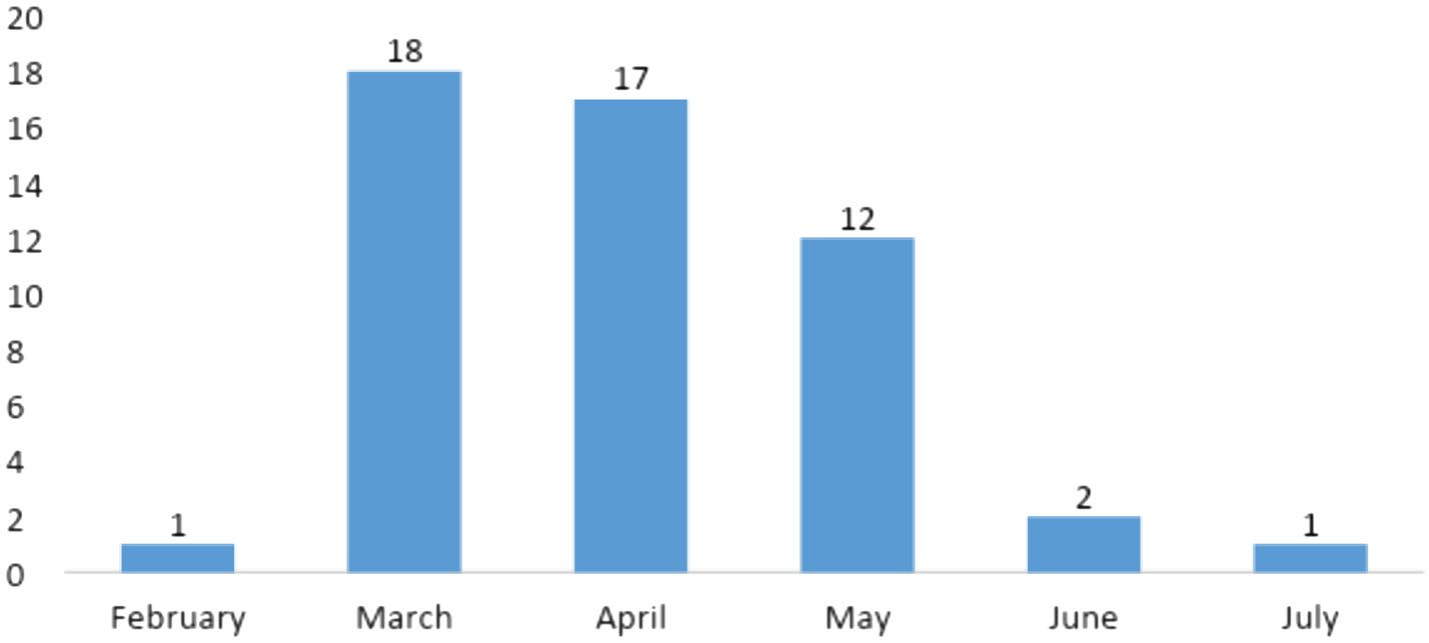
Evolution of the analyzed news between February and July (2020). The highest number of it was registered in March and April

In line with other academic works that used content analysis, such as Brammer and Millington (2006), Fernández-Gil (2010), Belso-Martínez et al. (2020), and Islam et al. (2021), we carried out a process of systematization of the data collected, which permitted an analysis of the frequencies, scope, and content of the press articles. The study's final performance indicates how much news has been published considering the month of publication, the firm's strategic response, and where it has been produced, thus allowing the starting point for the following discussion. The evolution by month for each analyzed news is showed in Fig. 3. It is noteworthy that March and April registered the highest number of information.

Related to the scope and content, we considered the type of newspaper and distinguished between news items from national newspapers (29 news items from 17 newspapers) and those published by regional newspapers (22 news from 15 newspapers). Among the national newspapers, prominent news items included four news articles from the newspaper El Mundo and three news items published by La Razón and Economía Digital. We analyzed other main news articles, including four news items from the newspaper Valencia Plaza and three news items posted by Las Provincias at the regional level.

Finally, the news content was interpreted using the Atlas.ti software program, the umbrella and relations defined and the recommendations proposed by Corbin and Strauss (2008). By carrying out this task, a total of 90 citations were obtained. When the relationship map was drawn, we then proceeded to classify and interpret it. As a result of these tasks, we noted that 25 citations reflected examples of the type of response in the Valencian Community. The remaining 65 citations were representative of the other regions in Spain (Table 1).

**Table 1:** Scope and content of the newspaper citations analyzed

	Valencian community	Rest of Spain	Total
Exit	0	3	3
Retrenchment	0	18	18
Persevering	1	5	6
Innovating	24	39	63
Total	25	65	90

Source: Own elaboration

After this analysis, we obtain two important conclusions. First, according to the evolution of the news, in Spain, the textile industry was on social media, especially during the three first months (March, April, and May) in which its firms were more involved in the challenge of creating products that could help medical and civil professionals, and under a situation characterized as highly complex (supply problems, homebound workers, and lacking technology). Second, we observed that 70% of the content referred to responses that can be classified as innovating (63), while the remaining 27 (30%) can be categorized out of this group. Specifically, 3 of them were exit, 6 persevering, and 18 retrenchment 20%. It is noteworthy that practically all of the regional news items and those from the Valencian community referred to innovating at the territorial level.

As a result of the content analysis, we can conclude that existed an uneven performance in the strategic response of the Spanish textile firms. According to the newspapers, firms belonging to the VTC battled against the coronavirus crisis, mainly implementing an innovator response. It means that when these firms were the object of public diffusion, it was to echo new products developed to combat the pandemic (masks, PPE, and other sanitary textile products) and a new process implemented and knowledge and research. At this point, the second question serves as a guide of this paper: the territorial or location-specific factors that influenced it. In the new part, we discuss it.

### Discussion on newspapers: the role of the location

Authors such as Chen (1996) argued that the result of any company's strategic response to a threat could be interpreted using a model termed AMC (awareness-motivation-capability). Awareness is understood as a necessary condition for any strategy, and it is based on the company's subjective interpretation of a particular situation (e.g., the pandemic and the lack of sanitary textile material); motivation refers to the company's desire to implement strategic responses to confront this situation (e.g., solidarity, opportunity, necessity) and capability can be understood as the company's ability to reorganize its resources and acquire those that are needed to act effectively (e.g., knowledge, certificates, cooperation). As we have discussed, as a result of this subjective analysis, firms can decide close (exit), lay-offs (retrenchment), not do nothing (persevering), or a related diversification through similar activities (innovation).

If we compare the newspapers informing about "Exit" and "Retrenchment" in response to the crisis, quotes are reflected in the rest of Spain and not in the Valencian Community. Quotes including the words "lay-offs, reducing (activity), costs, closing, exit, and bankruptcy were common on all of them. As example:

*The hotel industry and travel agencies will be the most affected, along with the textile industry in general, according to Cepyme. The coronavirus crisis severely affects most Spanish business fabric, which has seen almost all its activity paralyze overnight. Large companies have already begun to present an avalanche of ERTE to face losses and try to preserve the millions of jobs that are at stake, but SMEs and the self-employed are also being seriously harmed.*

(Heraldo de Aragón,—March 21, 2020).

*Experts are pessimistic about the coronavirus crisis's impact on the textile industry and anticipate a drop in their income close to 20%.*

(El Economista—April 6, 2020).

*The globalized supply of products and merchandise and the extended international production chains have increased the degree of vulnerability of a delocalized and decentralized economy ... Faced with this situation, sectors and citizens must move towards more local models ... greater flexibility, and adaptability to the dynamic and changing contexts that we face.*

(El Diario Vasco—April 20, 2020).

*Inditex, which until mid-afternoon anticipated a reduced schedule from 10:00 a.m. to 6:00 p.m. and extreme the cleaning and disinfection work for its network of stores in Spain -with temporary closure in the community transmission areas-, announced at the last minute the closure temporary of all of them "following the recommendation of the administrations." It implies the immediate closure of 1,500 establishments; the Cortefiel chain made the same decision. El Corte Inglés has decided to close down a part of its centers. Covid-19 catches the textile in what could be an excellent spring-summer campaign, with business on the rise due to high temperatures. Everything indicates that the collections will go almost entirely to the sales.*

(Faro de Vigo, March 14, 2020).

Meanwhile, precisely the opposite situations occurred in Valencian companies, even special cases such as a bankrupt company that voluntarily reopened its facilities to produce masks for the entire population of companies that redirect their production:

*Many of these companies have reoriented part of their production towards the manufacture of gowns or masks.*

(Economía Digital—April 19, 2020).

*A research group at the University of Alicante (UA) works with 3D printers to adapt diving masks to which only the filters would have to be changed to fit masks, goggles, and protective screens.*

(Informacion—March 18, 2020)

*Although the Castellón textile sector does not currently have too many companies ... the production rebound would be welcomed like "May water" ...we have the capacity to increase our textile production in Castellón by 30% more.*

(Candid Penalba former president of ATEVAL, Las Provincias—March 19, 2020)

*With a dry cleaning service, companies have machines to disinfect masks at more than 90 degrees of temperature… Any help, however symbolic it may be, is essential from the textile sector, which does not give up on the philanthropic work of everything, whatever is in your hand.*

(Valencia Plaza—March 20, 2020)

*In liquidation and without workers, the children's fashion company Confecciones Sulfy will reopen its facilities in the Valencian town so that volunteers can use its machinery to produce protective sanitary garments.*

(Expansion—March 30, 2020)

In line with the literature on business turnaround and crisis, the strategic response based on the exit, liquidation, or reduction of the activity is associated with interpretations of the crisis' causes as external and not controllable (Barker & Barr, 2002). Also, the decision to close or significantly reduce the activity, selling part of the assets, and restructuring the workforce can be due to a real situation of a substantial decline in the company's business (Rico et al., 2020). These aspects seem to have been the case suffered by other Spanish geographical areas (Gutierrez, 2021; Sio, 2020).

Related to the "Persevering" code that is part of the subcodes "supply chain," "market," and "new normal," practically all the news was published referring to textiles from the rest of Spain. In March, due to supply problems from Asian suppliers, Spain had severe problems sourcing raw materials such as yarns, fabrics, and semi-finished products that were key to producing sanitary textiles other textiles demanded by society and clients. Hence the call of the institutions to resist, look for other alternatives or wait for the fair guarantees to be obtained to start manufacturing was different in Spain than in the VTC:

*It is undoubtedly a fact that shows the weakness of the global supply chain due to the enormous dependence on China.*

(Marta Castells, general secretary of the Texfor textile, El Mundo—March 1, 2020)

*The shortage of basic protection material such as masks… The solution may lie in taking advantage of the textile industry of the province to manufacture these highly demanded items.*

(Gaceta de Salamanca—March 25, 2020).

*Due to relocations in the sector, only the most basic ones, the surgical ones, and not the filtering ones, can be manufactured in Spain.*

(Digital Economy—March 26, 2020).

*Our industry occupies a strategic place in the Spanish economy, and we cannot allow it to be vulnerable or dependent on external agents.*

(El Periodico de Aragón—March 26, 2020).

*Once the fabric is homologated, we can start manufacturing instantly.*

(Angel Asensio, president of the Spanish Federation of Garment Companies. Fedecon—Digital Economy, March 26, 2020).

*The Valencian textile industry will finally be able to manufacture surgical masks with all the guarantees.*

(Valencia Plaza—March 28, 2020).

Mueller et al. (2001) have argued that one of the main reasons to favor or disinhibit the reaction is how managers perceive the crisis. Suppose it is perceived as temporary or cyclical (for example, as crises in the textile sector have been in the past). In that case, efforts in innovation and reorientation will be much less or more conservative than if this is perceived as structural. Works such as that of Pla-Barber et al. (2007) show that this passive strategic response can be moderated by the experience and education of the managers and by family ownership of the company.

Finally, upon the innovating strategic response is remarkable that it was the one that the press most echoed. On the one hand, 63 of the 90 events analyzed had to do with the reorientation and diversification of textile businesses. On the other, 24 of the 25 news items referring to the TVC reported from different perspectives. Thus, on the one hand, the press release issued in mid-March by the main Spanish national association (Consejo Intertextil Español) highlighted, indicating that "the Spanish textile industry was at the service of the government" and reporting on the actions carried out such as information on the productive capacities, availability of raw materials and other resources to dedicate them to the production of sanitary textiles. And on the other, because he explicitly cited specific examples of such responses by companies in the territory (for example, Textils Mora, Textiles El Delfín, Rapife, Marie Claire, or Cotoblau).

Authors such as Staw et al. (1981) argue that a critical situation can produce two types of responses: one, more rigid or aimed at preserving resources. Another more innovative focused on the reorganization of activities and the search for solutions. This proposal connects with how the threat is perceived, and the capability to implement the response is valued. Therefore, if the importance of acting and being supportive is generally recognized, but there are informational asymmetries on how to do it, an uneven performance can be expected (Chen, 1996). Therefore, geographic proximity, the interaction between the various agents that make up the territory (clustering), and institutional support are crucial aspects of this reorganization and diversification process (Belso-Martinez et al., 2020; Puig et al., 2013). Some examples of this influence, the transmission of knowledge, and messages that supported the reorganization are the following:

*The associations that make up the CIE, ATEVAL, and TEXFOR have asked the companies to report on their productive capacities, raw materials, and resources … One hundred companies join the emergency.*

(Valencia Plaza—March 22, 2020).

*When the production of the new textiles is approved, we can start manufacturing immediately*.

(Angel Asensio, President of the Spanish Federation of Clothing Companies. Fedecon—Economía Digital—March 26, 2020).

*The collaboration of ATEVAL and AITEX has been crucial as it has been responsible for providing and facilitating companies that comply with certain hygiene conditions.*

(Valencia Plaza—March 28, 2020).

*The Municipality of Ontinyent has signed an agreement with the Association of Textile Entrepreneurs of the Valencian Community (ATEVAL) to launch an initiative to make sanitary textile a strategic industry.*

(El Mundo—April 14, 2020)

Moreover, other news related to institutional support and reduction of uncertainty about production processes would be the following:

*The Minister of Industry, Reyes Maroto, met telematically last Monday with the representatives of the leading industry associations in Spain, such as automobiles, textiles or equipment, to coordinate the manufacture of products with which to face the pandemic*.

(El Periódico de Aragón—March 26, 2020).

*The Spanish textile industry completes the production of up to 160.000 surgical masks a day to help cut the coronavirus contagion chain.*

(Economía Digital—March 26, 2020).

*The aim is not to completely transform our activity but to achieve a versatile industry that produces sufficient sanitary equipment when there are no epidemics; to be prepared to adapt to large volumes quickly, ensuring the country's supply in times of crisis*.

(Candid Penalba, former president of ATEVAL, El Mundo—April 14, 2020).

*The change in the Valencian textile has no way back, and politicians and businessmen continue to make steady progress in this project*.

(Valencia Plaza—May 13, 2020).

*In the week from March 31 to April 13, the state of alarm was released, and the cessation of activity was extended ... the textile industry was able to continue operating, having been put at the service of the Administration for the manufacture of sanitary material.*

*(*CE Noticias Financieras—May 28, 2020).

In short, after analyzing the content and scope of the written press, it is possible to obtain three key conclusions: (1) there is a coincidence between the months that have reported the most on the textile industry and the first months of the pandemic, (2) the articles published reported on the economic costs of the pandemic, but also on solidarity initiatives that textile companies were implementing (especially in the VTC) and (3) this propaganda served to guide and encourage public institutions in initiatives for change as well as to coordinate the different agents towards a single objective.

**Fig. 4:**
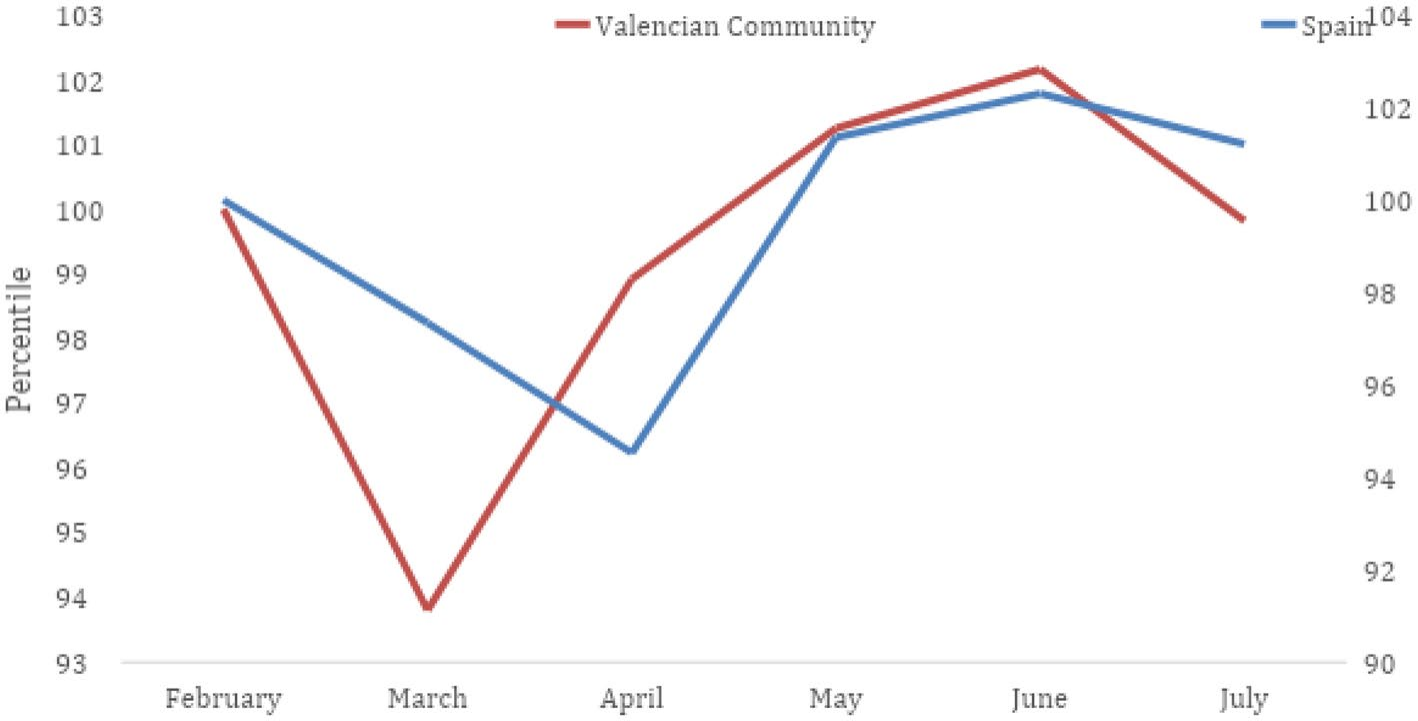
Evolution of the affiliation of workers in the Spanish textile-clothing industry. Source: Own elaboration from Generalitat Valenciana (2020)

The question regarding why the VTC behaved differently from other Spanish textile territories lies in two main aspects. First, the productive tradition of that place in home-technical textiles (easier to convert to sanitary materials than other subsectors such as knitwear, garments, or wardrobe). And second, be part of a textile cluster in which all its actors interacted effectively to facilitate the challenge of fighting the challenge of the pandemic and reducing the impact of the crisis. The Annex 1: Fig. 4 includes the figure that collects the evolution of employment in the Spanish textile industry as a percentage. In this figure, a heterogeneous response is observed, and that at first (March), the VTC was more intense, but after (April and May) the recovery of the situation faster.

## Conclusions

The main objective of this work was to analyze the strategic response of the VTC's firms to the coronavirus crisis and the territorial factors that influenced it. For this, we analyzed the content of press articles published during February–July of 2020 in leading Spanish newspapers. We obtained a total of 91 citations that highlighted the strategic response of textiles firms to this crisis (i.e., retrenchment, persevering, innovating, and exit).

This analysis revealed that, as happened in the fashion industry (Kim & Woo, 2021), the Spanish textile industry responded heterogeneously. At the national level, most firms implemented retrenchment strategies. While at the regional level (Valencian Community), organizations that formed part of the so-called VTC developed innovative strategies, which helped combat the pandemic. The factors and capabilities that influenced this response were structural and circumstantial (Ambrosini & Bowman, 2009).

From the theory of the dynamic capabilities (Teece, 2007), it could be argued that the shared resources of the location influenced the strategic response of companies improving their abilities and potential to detect and capture opportunities and solve problems (Barreto, 2010). The origin of these shared resources is a deep-rooted tradition in producing technical-home textiles and one organizational model forming a cluster that provided valuable information and knowledge (structural). Moreover, we also observed rapid acts of solidarity and strong cooperation between all of the territory stakeholders (institutions, associations, and government) favored by the echo they were having in the press (circumstantial).

Given the complexity of the current international competitive scenario and the risk of unexpected future events, three essential lessons can be drawn: (1) At a strategic level about having little control over the supply and value chain (Gereffi, 2020). (2) Regarding the location-specific and competitive advantages that proximity and clustering can provide to businesses (information, knowledge, trust, flexibility) (Fromhold-Eisebith et al., 2021). (3) For politicians and on the strategic value of the textile-clothing sector, particularly for society and the economy.

This study has certain limitations, such as the fact that we focused on a single region (i.e., Valencia) and used Atlas.ti to obtain and codify press news. Future research could replicate this study in other contexts and analyze it at the company level to identify the dynamic capabilities critical to confronting an unexpected crisis (sensing, seizing, and reconfiguration) and its effects on the structure of the territory (Schilke et al., 2018).

## Data Availability

Data analyzed in this study were a re-analysis of existing data, which are openly available at locations cited in the reference section. Further documentation about data processing is available at Sects. 3 and 4.

## Annex

See Table 2  and Fig. 4.

**Table 2:** Relationship between codes and subcodes applied in ATLAS.Ti

Temporary code	Relationship	Codes (Wenzel et al., 2020)	Relationship	Subcodes	Relationship	Spatial code
*Strategic response of the Textile Industry to Covid19*	It is associated with	Retrenchment	It's part of	Lay-Off Reducing Costs	It is a property of	Spain
		Persevering		Supply Chain Market New Normal		
		Innovating		Product Process Knowledge		Valencian Community
		Exit		Closing Exit Bankruptcy		

Source: Own elaboration
